# Individual yeast cells signal at different levels but each with good precision

**DOI:** 10.1098/rsos.241025

**Published:** 2025-04-30

**Authors:** Steven S. Andrews, Roger Brent

**Affiliations:** ^1^ Bioengineering, University of Washington, Seattle, WA, USA; ^2^ Basic Sciences, Fred Hutchinson Cancer Research Center, Seattle, WA, USA

**Keywords:** systems biology, cell signalling, cell biology modelling, information theory

## Abstract

Different isogenic cells exhibit different responses to the same extracellular signals. Several authors assumed that this variation arose from stochastic signalling noise with the implication that single eukaryotic cells could not detect their surroundings accurately, but work by us and others has shown that the variation is dominated instead by persistent cell-to-cell differences. Here, we analysed previously published data to quantify the sources of variation in pheromone-induced gene expression in *Saccharomyces cerevisiae*. We found that 91% of response variation was due to stable cell-to-cell differences, 8% from experimental measurement error, and 1% from signalling noise and expression noise. Low noise enabled precise signalling; individual cells could transmit over 3 bits of information through the pheromone response system and so respond differently to eight different pheromone concentrations. Additionally, if individual cells could reference their responses against constitutively expressed proteins, then cells could determine absolute pheromone concentrations with 2 bits of accuracy. These results help explain how individual yeast cells can accurately sense and respond to different extracellular pheromone concentrations.

## 1. Introduction

Cell signalling systems sense extracellular conditions and transmit information about them into the cell. The cell then uses the information to make decisions, such as whether to grow, undergo apoptosis, or differentiate. Incorrect decisions can lead to undesirable outcomes, so one would reasonably expect that cell signalling systems would have evolved to transmit information accurately [1,2]. Indeed, yeast cells (*Saccharomyces cerevisiae*) reliably select the nearby mating partner that produces the strongest pheromone signal [3], which demonstrates that they accurately sense, transmit and interpret information about external pheromone concentrations.

However, the amount of information transmitted through cell signalling systems has not been quantified until recently, when several groups of researchers applied agonists to isogenic populations of mammalian cells and measured the responses by individual cells [4–7]. They found wide variation, which they quantified to show that the channel capacity for a single cell, defined as the maximum information that can be discerned about the stimulus level from one cell’s response [8–17], was only about one bit. This number corresponds to two states, which implies that a cell’s response was adequate for determining whether the agonist was present or not, but could not give further detail about its concentration. These observations led to suggestions that single cells could not sense the extracellular environment precisely, so populations of cells might need to combine information from multiple sources to make correct decisions [4,18,19].

These observations are also consistent with a different interpretation, in which variable responses from single cells do not arise from noisy signalling within cells but from pre-existing differences between cells [20–24]. In this interpretation, which agrees with substantial prior work by ourselves and others [25–29], individual cells behave differently from each other, but each is able to distinguish between different external conditions reasonably precisely by itself. This view would explain how individual cells might achieve accurate responses from single inputs. It is also consistent with the possibility that populations of cells could combine information from multiple sources to make even better decisions [23].

The work presented here extends this latter view. We infer channel capacity from single-cell time series data in *S. cerevisiae* using a new data analysis approach. We find, in agreement with prior reports, that the measured channel capacity for the response of a randomly chosen single cell is only about one bit. However, quantification of the signalling noise within individual cells reveals that intracellular channel capacities are often over 3 bits. We also quantify the relative contributions of signalling system noise, gene expression noise, and pre-existing cell-to-cell variation in signalling and gene expression. We find that, in our data, cell-to-cell variation represents 91% of the total variation, experimental measurement error represents 8% of the total variation and noise that originated within the signalling pathway is under 1%. Together, these results indicate that yeast cells are able to transmit signals with good fidelity.

## 2. Methods

### 2.1. Experimental data

**Figure 1.**
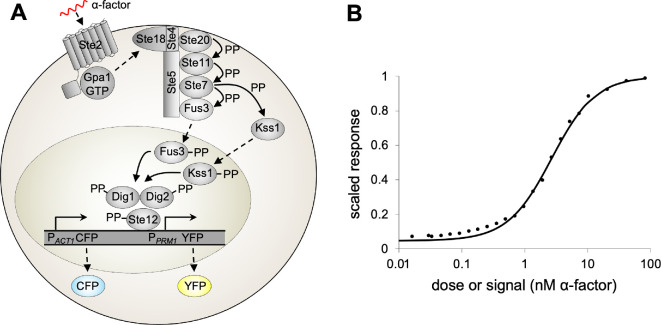
(A) The yeast PRS in mating type a cells. Pheromone (α-factor, in red) binds to G-protein coupled receptors (Ste2), causing dissociation of heterotrimeric G-proteins (Gpa1/Ste18/Ste4), which recruits Ste5 scaffold proteins to the cell membrane and induces signalling through a MAP kinase cascade (Ste11, Ste7, Fus3 and Kss1); the MAP kinases activate Ste12 transcription factors which then promote expression of pheromone-responsive genes, in this case by binding to the *PRM1* promoter which then leads to YFP expression [30]. Meanwhile, constitutive expression at the *ACT1* promoter leads to constant RFP or CFP expression (only CFP is shown here). The pheromone concentration is the signal, YFP fluorescence is the pheromone-induced response, and CFP fluorescence is the constitutive response. (B) Dose-response curve for the PRS, measured 3 h after stimulation. Points represent experimental data from [1]. and the line represents a Hill function fit to the data from equation (2.1) and [31]. Points and fit were scaled to approach a maximum of 1.

Haploid yeast cells have two mating types, MATa and MATα. The MATα cells secrete α-factor mating pheromone, which the MATa cells detect and may act upon, which can then lead to mating. The signalling system within the MATa cells (figure 1A), called the yeast pheromone response system (PRS), transmits information about α-factor binding at cell-surface receptors to the cell nucleus. It is a prototypical G-protein signalling system, bears close homology to many mammalian signalling systems and has been studied thoroughly by many researchers, including ourselves [1,25,31–34]. See reviews [30,35–37] for details about this system.

Once α-factor binding information reaches the cell nucleus, it induces the expression of several genes that initiate progress toward mating. In addition, the cells that we investigated were engineered to express yellow fluorescent protein (YFP) from a pheromone-responsive promoter ($P_{PRM1}$) and either red or cyan fluorescent protein (RFP or CFP) from a constitutive promoter ($P_{ACT1}$) [1,25] (electronic supplementary materials, section 1 (SM-1) presents genetic and experimental details). These reporter proteins were not intended to represent specific native yeast proteins but were simply used to enable the quantification of pheromone-induced and constitutive expression rates. YFP and CFP maturation half-life times were found to be about 39 and 49 min, respectively, while degradation was negligible [38].

We used two previously published data sets in our analysis, from essentially identical cells. The first, from [1] and shown in figure 1B, represents the dose–response function for the PRS, measured 3 h after pheromone stimulation. It was collected using flow cytometry in which each cell’s response was computed as the ratio of YFP (pheromone-induced) fluorescence to RFP (constitutive) fluorescence in the same cell. Defining the response as this ratio automatically corrected for brightness variation in the exciting light source. It also corrected for cell-to-cell variation in overall protein expression rates (described elsewhere [25] and below as ‘expression capacity’), in order for the response to accurately represent the relative level of pheromone induction. These values were averaged over many cells.

We required a dose-response function that was defined everywhere rather than only at discrete pheromone concentrations, so we based our analysis on a Hill function that we had fit to these experimental data [31] rather than the raw data themselves. This fit is the four-parameter Hill function


(2.1) $$\bar{r}(s)=B+A\frac{s^{N}}{E^{N}+s^{N}}$$

where $\bar{r}(s)$ is the mean response over all cells as a function of the signal, s is the signal or pheromone concentration, B is the baseline, A is the Hill function amplitude, E is the ${EC}_{50}$ (the signal value that produces half-maximal response), and N is the Hill coefficient. We scaled the experimental data so that the fitted response would asymptotically approach a maximum value of 100% (i.e. A+B=1.0). We also constrained the fit so that the baseline would equal the response that arose with no pheromone addition, which was 4.7% of the maximal response. Best fit values are B=0.047, A=0.953, E=2.67 nM and N=1.24. This fit has a root mean square (r.m.s.) error of 0.18%, showing excellent agreement with the data (figure 1B).

These experimental data, and the Hill function fit to them, represent the dose–response curve for the cell population, and not for individual cells. Such a population-average dose–response curve is necessarily less steep than the dose–response curves for the individual cells due to what has been called the ‘response diversity effect’ [23]. It arises from the fact that the dose–response curves for individual cells have different EC_50_ values, which then causes the average of these curves to exhibit a more gradual transition between low and high responses. We do not have single-cell dose–response curves, so did not correct for this effect. We discuss it more below.

**Figure 2.**
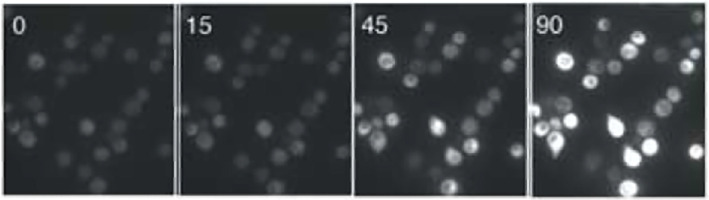
Microscope image of YFP fluorescence from several cells at 0, 15, 45 and 90 min after measurement began (which was 10 min after pheromone addition). Note that the brightest cells at 90 min were also the brightest cells earlier, showing temporally consistent variation. Copied with permission from [25].

The second dataset, described in [25,39], consists of inverted epifluorescence microscope images of single-cell responses to pheromone stimulation. Figure 2 shows a set of example images. These cells were arrested in either the G1 or G2 cell cycle states, using a kinase inhibitor that acted on a mutant Cdc28 cell cycle kinase (cdc28-as2). They were also exposed to one of 5 time-invariant concentrations of pheromone. At each of these concentrations, the image data capture the YFP and CFP fluorescence intensities of roughly 100 individual yeast cells at 14 equally spaced time points after pheromone addition. We minimally filtered the image data to remove entries for dead cells, badly segmented cells and outlier measurements as recommended by Bush *et al*. [39] (SM-2 describes this filtering). We then integrated the images over the cell areas to determine YFP and CFP fluorescence intensities.

**Figure 3.**
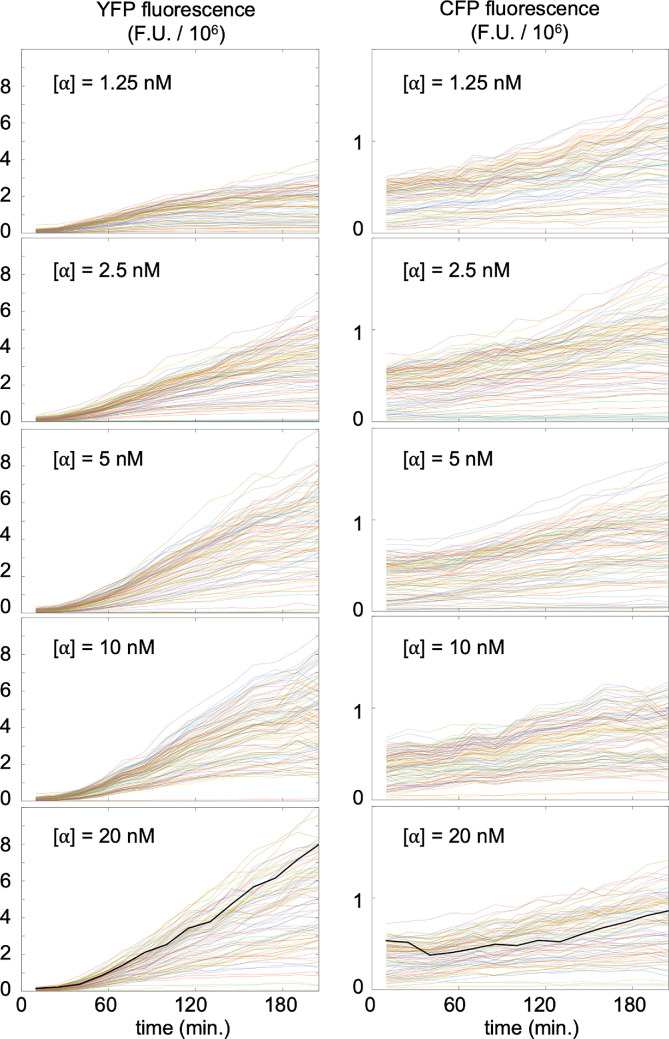
Left: YFP fluorescence from individual cells over time after pheromone addition, quantified in arbitrary fluorescence units (F.U.). Right: CFP fluorescence, expressed from a constitutive promoter, from the same cells. Data for these panels were measured for [25] and made publically available in [39].

Figure 3 shows the resulting YFP and CFP fluorescence intensities, for each pheromone dose level, where each line in a graph represents fluorescence from a single cell. The graphs in the left column show that YFP fluorescence, from pheromone-responsive promoters, stayed low initially and then increased nearly linearly over time for at least 3 h. Neither pheromone nor intracellular YFP was degraded significantly during these experiments, implying that the PRS within each cell must have signalled at a nearly constant rate [25]. The graphs in the right column show that the CFP fluorescence, from constitutively expressed promoters in the same cells, did not respond to pheromone stimulation but simply increased due to protein accumulation. The black lines in the bottom graphs highlight the YFP and CFP fluorescences for a randomly chosen cell.

### 2.2. Theory background

Information theory is an appropriate approach for quantifying biological signalling fidelity because it directly addresses a critical question, which is how much information can actually be transmitted through the signalling pathway [8–17]. It is quantified as mutual information, which represents how much information is shared between the signal and response. Equivalently, the mutual information represents how much one can learn about the signal when being told what the response is. For simplicity, we focus on a single snapshot of the system, meaning that the signal is simply the external pheromone concentration, which is time-invariant, and the response is the amount of YFP fluorescence from a single cell at a particular moment (note that this snapshot necessarily includes some intrinsic signal averaging because fluorescent proteins do not mature at a fixed time after their expression, but with a spread of times [38]). The mutual information is given by the equation


(2.2) $$I(s;r)=\int_{s}\int_{r}p(r|s)p(s)\log_{2}\frac{p(r|s)}{p(r)}\text{d}r\text{d}s,$$

where p(s) is the signal distribution, meaning the probability that the input signal has the given value and p(r|s) is the conditional probability of observing response r for given signal s. The p(r) function is the response distribution, which represents the probability of observing the given response while integrating over all input signals,


(2.3) $$p(r)=\int_{s}p(r|s)p(s)\text{d}s.$$

The mutual information is quantified in bits (due to the base 2 logarithm in equation (2.2)), with the interpretation that $2^{I(s;r)}$ represents the number of signal values that can be reliably distinguished from a given response.

The natural signal distribution, here meaning the pheromone concentrations that yeast cells are actually exposed to in their natural environments, is unknown. To address this, we follow the convention by optimizing p(s) to yield the maximum possible mutual information [9], which is then called the channel capacity. The channel capacity represents the upper limit to the amount of information that is actually transmitted in natural situations. We performed this optimization numerically with the Blahut–Arimoto algorithm [9,40], an iterative approach that optimizes p(s), using a known p(r|s) function, in order to find the maximum possible mutual information, I(s;r). We describe our implementation of it in the Appendix.

A separate approach for investigating signalling fidelity looks at where the variation comes from [41]. This approach was first used by Elowitz *et al*. [42], who defined ‘intrinsic noise’ as variation that arises from the discrete nature of the gene expression process and ‘extrinsic noise’ as variation that is global to a single cell but varies from one cell to another (this latter variation may be time-dependent). In Colman-Lerner *et al*. [25], we extended this approach to variation in signalling-induced gene expression in *S. cerevisiae* by decomposing the multiple contributions of extrinsic noise. In that work, we modelled the fluorescence of cell i at time ΔT after pheromone stimulation as


(2.4) $$\begin{array}{lcr} & y_{i}=P_{i}\Delta T\times E_{i}, & \end{array}$$

where $P_{i}$ is the ‘power’ of the signalling pathway, which is a function of both the pheromone input concentration and the ability of the pathway to transmit signals, and $E_{i}$ is the ‘power’ of the cell’s gene expression system. These two power terms are subdivided into values that are consistent over time for a single cell but might vary between individual cells and values that vary stochastically over time,


(2.5) $$\begin{array}{lrlr} & P_{i} & =L_{i}+\lambda_{i} & \\ & E_{i} & =G_{i}+\gamma_{i}. & \end{array}$$

Here, $L_{i}$ is called the ‘pathway capacity’, $\lambda_{i}$ is the ‘pathway noise’, $G_{i}$ is the ‘expression capacity’ and $\gamma_{i}$ is the ‘expression noise’. The $L_{i}$, $\lambda_{i}$ and $G_{i}$ terms quantify different sources of ‘extrinsic noise’, while $\gamma_{i}$ is the same ‘intrinsic noise’ that was defined in [42]. Note that references to ‘cell-to-cell variation’ in the present work refer to temporally stable differences, meaning the combination of the $L_{i}$ and $G_{i}$ terms.

The total YFP fluorescence variation for a population of cells arises from variation in each of these terms. Using η as the coefficient of variation, defined as the ratio of the standard deviation (s.d.) to the mean, the total variation is


(2.6) $$\begin{array}{lcr} & \eta^{2}(y)=\eta^{2}(L)+\eta^{2}(\lambda )+\eta^{2}(G)+\eta^{2}(\gamma )+2\rho (L,G)\eta (L)\eta (G), & \end{array}$$

where terms within parentheses indicate the varying components. The derivation of this equation is described in detail in the electronic supplementary material to [25]. In brief, the squared coefficient of variation for a product of two independent random variables, here $P_{i}\times E_{i}$, is the sum of their squared coefficients of variation. Also, the variance of the sum of two independent random variables, here $L_{i}+\lambda_{i}$ and $G_{i}+\gamma_{i}$, is the sum of their variances; after some algebraic manipulation, and the assumptions that the mean values of $\lambda_{i}$ and $\gamma_{i}$ equal zero, these sums also become sums of squared coefficients of variation. Together, these yield the first four terms of equation (2.6). The final term in the equation accounts for correlations between the pathway capacity, L, and the expression capacity, G; we assumed previously that this term can be ignored [25], and do so here as well.

## 3. Results

### 3.1. Population channel capacity is 1.35 bits

**Figure 4.**
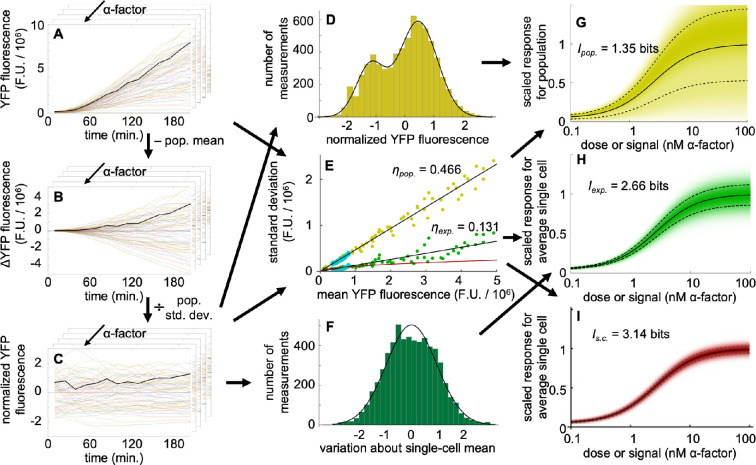
Channel capacity computation method. (A) Filtered single-cell YFP fluorescence data, where each line represents fluorescence from a single cell over time; points represent y(s,i,t). A representative cell is shown here and in the two following panels in black. Each layer represents a different pheromone dose. (B) The same data, but with the population mean (all cells at a given time point and pheromone dose, equation (3.1)) fluorescence values subtracted. (C) The normalized single-cell data, in which fluorescence difference values were divided by the population standard deviation; points represent $\tilde{y}(s,i,t)$ from equation (3.3). (D) Distribution of normalized fluorescence values from panel C. The line is a best fit using a sum of two Gaussians. (E) Correlation between mean and s.d. values for the cell population in yellow equations (3.1) and (3.2) and for an average single cell in green equations (3.1) and (3.7). Cyan points represent CFP (constitutive) fluorescence. Each point represents a single time point and pheromone concentration. Black lines are best fits to the yellow or green points and were constrained to intersect the origin. The red line represents only signalling and expression variation, from the fit presented in equation (3.9). (F) Distribution of normalized fluorescence values about the normalized single-cell mean values, scaled with the single-cell s.d.; this is $\tilde{\tilde{y}}(s,i,t)$ from equation (3.8). The line is a best fit with a single Gaussian. (G) Signal–response–variation (SRV) diagram for the cell population. Shading represents the conditional response probability, p(r|s), the solid line represents the mean of this distribution, r(s), and the dashed line represents the s.d. of this distribution. (H) SRV diagram for an average single cell, showing lower variation and hence greater information transfer; as described in the main text, the variation shown is likely dominated by experimental fluctuations. (I) SRV diagram for an average single cell, now with variation that only represents signalling and expression noise; the low variation enables high information transfer.

We quantified the signalling channel capacity in three ways (analyses were performed using Mathematica; source files are in electronic supplementary material). The first, shown with yellow shading in figure 4, evaluates the total observed variation in response among the cells that comprise the population. This allows a meaningful comparison with prior results [4–7].

From the single-cell data for YFP fluorescence that are shown in the left column of figure 3 and illustrated in figure 4A, we computed the mean and standard deviation YFP fluorescence across the cell population at each time point and pheromone dose (14 time points × 5 doses = 70 data points). Defining y(s,i,t) as the YFP fluorescence value for pheromone level s, cell number i, and time point number t, these population mean and population standard deviations are, respectively


(3.1) $$\mu (s,t)=\langle y(s,i,t)\rangle_{i}$$

(3.2) $$\sigma_{pop.}(s,t)=\sqrt{\langle [y(s,i,t)-\mu (s,t){]}^{2}\rangle_{i}}.$$

Angle brackets denote a mean over the variable that is listed in the subscript.

A scatter plot of these results, shown with yellow points in figure 4E, shows a linear relationship. This implies that the coefficient of variation, η, is constant between the mean responses of the cell population and the variation across the population, independent of both pheromone dose and exposure time (here, $\eta_{pop.}=0.466$). The linear relationship agrees with prior experiments that investigated high-abundance proteins and was shown to be the expected outcome in cases where variation is dominated by extrinsic noise [25,43,44] (supposing only cell-to-cell variation, the outputs of a collection of cells would all scale to higher or lower values together when the cells are induced to express proteins at different rates, so the population standard deviations and means would also scale together). We also computed mean and standard deviation CFP fluorescence values in the same manner, shown in the same figure with the upper row of cyan points, and found the same linear relationship and a nearly identical coefficient of variation, now with $\eta_{C,pop.}=0.443$. This similarity is consistent with the variation in both YFP and CFP fluorescence arising primarily from variation in gene expression capacity [25], which is a cell-wide property. Both η values are comparable to values found previously, which range from about 0.2 to about 0.7 [5,25,43].

Next, we normalized the single-cell data by subtracting the population mean at each time point (figure 4B) and then dividing by the population s.d. at each time point (figure 4C). As an equation, the normalized data values are


(3.3) $$\tilde{y}(s,i,t)=\frac{y(s,i,t)-\mu (s,t)}{\sigma_{pop.}(s,t)},$$

where the tilde denotes normalization. Each of these normalized fluorescence values represents the brightness of a particular cell relative to the population as a whole, at any given time point. By construction, these values have a mean of 0 and a s.d. of 1. They exhibit a bimodal distribution, shown in figure 4D, likely arising from cells that were arrested in either the G1 or G2 cell cycle states [25,45]. While this figure combines normalized fluorescence values from all five dose values and 14 time points, we also created separate histograms for each dose value and for each time point; there were no significant differences between those histograms, or between those and the cumulative one shown in figure 4D. In addition, the comparable histograms for the normalized CFP fluorescence values exhibited similar bimodality. These results agree with expectations because cells arrested in G2 have twice as many fluorescent reporter genes. While it is possible to design experiments for detecting cell state directly with microscopy methods [46], those approaches were not used in the experiments discussed here. As a result, it is impossible to independently classify particular cells in this study as being in the G1 or G2 phases.

We combined several of the prior results to compute what we call a ‘signal–response–variation’ (SRV) diagram, shown in figure 4G. Here, the mean response is the steady-state dose–response curve from equation (2.1) and figure 1B, the response standard deviation comes from the $\eta_{pop.}$ value of 0.466 shown in figure 4E, and the shape of the response distribution at any given pheromone concentration is the bimodal distribution of YFP fluorescence values shown in figure 4D. Shading in the SRV diagram represents the probability of observing response r given that the signal is s, which is the conditional response probability, p(r|s). We computed the channel capacity from this conditional response probability using equation (2.2) and the Blahut–Arimoto algorithm (appendix).

We found that the channel capacity for the SRV diagram in figure 4G, which represents the total variation in response among the cells that comprise the population, is 1.35 bits; we call this the ‘population channel capacity’. This means that knowledge of the YFP fluorescence from any randomly chosen single cell is sufficient to convey 1.35 bits of information about the pheromone concentration. This corresponds to about 2.5 different states ($2^{1.35}$), so knowing the YFP fluorescence from one cell can generally indicate whether pheromone is present or absent but does not give much more detail than that. This low channel capacity value agrees with the about 1 bit that was found in studies of mammalian cells [4–7].

### 3.2. Measurement channel capacity is 2.66 bits

To remove the effects of cell-to-cell variation from other sources of variation, we assumed that the different slopes of the data traces shown in figures 3 and 4A arose from temporally stable cell-to-cell variation and that the small ‘wiggles’ within each of these traces represented a combination of single-cell noise and measurement error. This meant that we could quantify the noise amounts by determining the sizes of the wiggles.

In principle, these wiggle sizes could be computed by fitting smooth lines to the YFP fluorescence data and then computing residuals from them. However, such an approach would introduce artifacts from the necessarily imperfect fits to the non-linear time dependence. In more detail, fitting a straight line to each cell’s time-dependent response would introduce artifacts because the responses actually increase non-linearly, and fitting more complex functions would create a risk of overfitting. Thus, we chose a less direct but also less biased approach.

**Figure 5.**
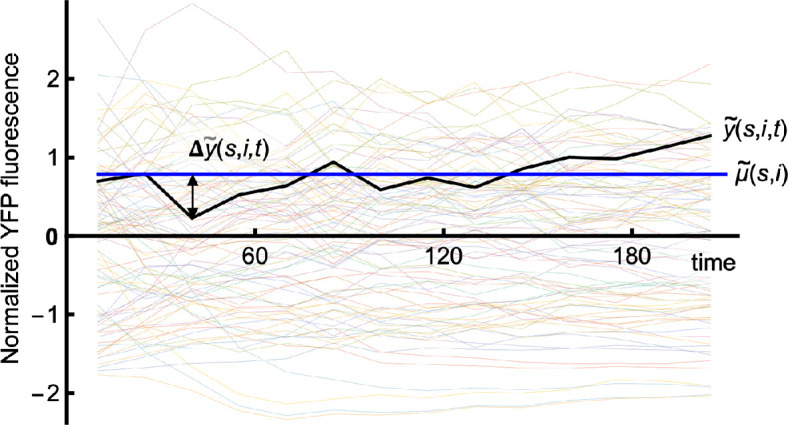
Normalized single-cell responses, shown for YFP fluorescence after addition of 20 nM pheromone. The black line shows the normalized response for a particular cell, $\tilde{y}(s,i,t)$ and the blue line shows its normalized mean value, $\tilde{\mu }(s,i)$.


Starting with the normalized data (equation (3.3) and figure 4C), we computed the normalized mean value for each cell over time,


(3.4) $$\begin{array}{lcr} & \tilde{\mu }(s,i)=\langle \tilde{y}(s,i,t)\rangle_{t}, & \end{array}$$

and then the difference between each normalized data point and this normalized mean value (see figure 5),

(3.5) $$\begin{array}{lcr} & \Delta \tilde{y}(s,i,t)=\tilde{y}(s,i,t)-\tilde{\mu }(s,i). & \end{array}$$

Then, at each time point and pheromone dose, we computed the rms average of these normalized variation values over all cells,


(3.6) $${\tilde{\sigma }}_{exp.}(s,t)=\sqrt{\langle \Delta {\tilde{y}}^{2}(s,i,t)\rangle_{i}}.$$

The ‘exp.’ subscript indicates that this is for experimental measurement error, as explained below. Finally, we removed the normalization by multiplying each rms average variation value by the population s.d.,


(3.7) $$\sigma_{exp.}(s,t)=\sigma_{pop.}(s,t){\tilde{\sigma }}_{exp.}(s,t).$$

The result is an average of the variation amount (or wiggle size) over all cells, at each time point and pheromone dose, in units of YFP fluorescence. Figure 4E shows the results with green dots, plotting these single-cell standard deviations against the mean fluorescence values. It again shows a linear relationship between the mean and standard deviations, now with a coefficient of variation equal to $\eta_{exp.}=0.131$. This single-cell value is smaller than the population one because it does not include cell-to-cell variation. Analysing the CFP data in the same way produced the lower row of cyan dots in figure 4E and a similar coefficient of variation of $\eta_{C,exp.}=0.104$.

We also computed the distribution of variation about the respective single-cell mean values. To account for the fact that brighter cells exhibited more variation (in figure 5, note that the cells near the top of the graph exhibit more variation than those near the bottom), we divided the normalized variation values by the normalized single-cell mean values,


(3.8) $$\begin{array}{lcr} & \tilde{\tilde{y}}(s,i,t)=\frac{\Delta \tilde{y}(s,i,t)}{\tilde{\mu }(s,i)}. & \end{array}$$

This yielded variation values that could be compared between different cells, as well as between different time points and pheromone doses. Figure 4F shows that the variation value distribution agrees reasonably well with a Gaussian, likely indicating that these variations arise from a sum of many factors (the Central Limit Theorem shows that a sum of independent random variables approaches a Gaussian distribution).

Figure 4H shows the SRV diagram for this variation. It assumes (i) that each cell’s dose–response curve matches that of the population average, given in equation (2.1), (ii) each cell’s variations over time are Gaussian distributed based on figure 4, and (iii) these variations have a coefficient of variation of $\eta_{exp.}=0.131$ from figure 4E. From it, we computed a channel capacity of 2.66 bits.

Our original interpretation of this value was that it represented the amount of information transmitted from an average cell’s pheromone receptors to its induced protein expression (corresponding to the effects of the $\lambda_{i}$ and $\gamma_{i}$ terms from equation (2.5)). However, the linear relationship between single-cell s.d. and mean values that is shown in figure 4E was unexpected because intrinsic noise standard deviations have been shown to scale as the square root of the mean, not in direct proportion to the mean [42–44]. Moreover, we realized that *any* noise that arises from a memoryless steady-state process, which is likely to be a good description for both the signalling pathway and gene expression in this experiment, *must* exhibit a square root relationship between the mean response and the standard deviation for a reporter that accumulates over time. This is because this response represents the sum of independent and identically distributed random values, and the standard deviation of such a sum always increases as the square root of the value (from the Central Limit Theorem).

**Figure 6.**
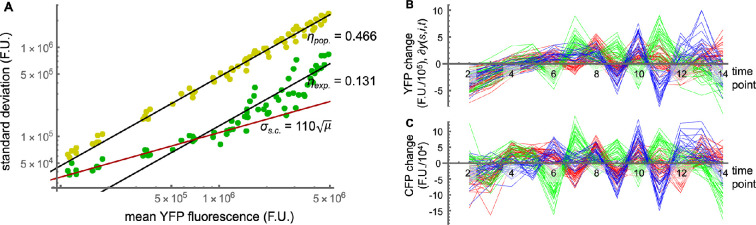
(A) Relationship between mean and standard deviation YFP fluorescence for population data (yellow) and single-cell data (green), shown on log-log scales. (B) and (C) Microscope artifacts for YFP and CFP.

We looked for the expected square root dependence by replotting figure 4E with log–log scales, which is shown in figure 6A. Indeed, this graph shows a square root relationship for the single-cell data points that represent low YFP expression (arising from low pheromone doses and/or short times after stimulation), and the previously observed linear dependence for single-cell data points for high YFP expression. Based on the above arguments, we believe that the region with the square root dependence represents signalling and expression noise in individual cells and that the region with the linear dependence arises from a different source. The only reasonable candidate for this other source is experimental fluctuations in the microscopy and image analysis.

Consistent with this identification, we observed variation in the data that clearly arose from experimental errors. Figure 6B,C shows fluorescence time-difference values, defined as


$$\delta y(s,i,t)=y(s,i,t_{j})-y(s,i,t_{j-1}),$$

for the YFP and CFP data for cells measured at 20 nM α-factor, with one line for each cell. These lines are colour coded to represent the three different microscope positions that were used while collecting these data. Clearly, the fluorescence differences are highly correlated for different cells that are at the same microscope position, and also the YFP and CFP data show the same trends. For example, the blue traces are all high for both YFP and CFP at time point 10 and all low at time points 9 and 11; these effects undoubtedly arose from changes in the microscope sensitivity. As one possibility, these results may have arisen from fluctuations in the brightness of the microscope’s exciting light source. We tried to remove these correlations from the data in preliminary work, but doing so led to additional artifacts and did not affect the results substantially, so they are not removed for the results presented here.

Thus, we believe that the channel capacity value of 2.66 bits represents the channel capacity of the measurement process itself, comprising both microscopy and image analysis. In this interpretation, the measurement process is a communication channel that has a physical cell as its input and a fluorescence value as its output, along with multiple sources of variation that disrupt perfect communication. Our result shows that these experimental methods had sufficient precision to reliably distinguish between about 6 different single-cell brightness levels, but not better than that. The linear relationship between the single-cell standard deviation and mean values implies that this measurement channel capacity is essentially the same for all cell brightnesses (this agrees with the expected result for a fluctuating excitatory light source, using similar logic as was given before for ‘extrinsic noise’). While this channel capacity result doesn’t convey any information about the cells being studied, it does illustrate the importance of high precision experimental methods.

### 3.3. Single cell channel capacity can exceed 3 bits

The square root dependence shown figure 6A represents the signalling and expression noise ($\lambda_{i}$ and $\gamma_{i}$) within single cells, not including cell-to-cell variation or measurement errors. We used it to compute the intracellular signalling precision.

The best-fit line to the square-root dependence is


(3.9) $$\sigma_{s.c.}(s,t)=(110{\text{ F.U.}}^{1/2})\sqrt{\mu (s,t)}.$$

Computing the channel capacity was more complicated than before because variation in the SRV diagram depended on the actual values of responses this time, and could not be computed from scaled values. To address this, we defined an ‘average cell’, indexed as $i_{av.}$, as one whose mean response is equal to the population average and whose variation over time is equal to the rms average variation. As equations,


(3.10) $$\begin{array}{l} \mu (s,i_{av.},t)=\mu (s,t), \end{array}$$

(3.11) $$\begin{array}{l} \sigma (s,i_{\text{av}.},t)=\sigma_{\text{exp}.}(s,t). \end{array}$$

We also assumed that this average cell was measured at the final time point (number 14, at 205 min after pheromone addition). We fit a Hill function to the population mean at the final time point, μ(s,14), to create a dose–response function with absolute units [31]; then extrapolating it to high doses showed that the average cell’s response at high pheromone concentrations was $\mu_{max}=4.83\times 10^{6}\text{ F.U.}$ With this, we rescaled equation (3.9) to


(3.12) $$\frac{\sigma_{s.c.}(s,t)}{\mu_{max}}=\frac{\left(110{\text{ F.U.}}^{1/2}\right)}{\sqrt{\mu_{max}}}\sqrt{\frac{\mu (s,t)}{\mu_{max}}}\approx 0.050\sqrt{\frac{\mu (s,t)}{\mu_{max}}}.$$

This enabled us to plot the SRV diagram for this ‘average cell’, shown in figure 4I. As usual, the mean dose–response curve represents the population average and is from equation (2.1). However, the variation in this case comes from equation (3.12), where $\mu_{max}$ is the maximum value of the dose–response curve. We again assumed Gaussian distributed noise, based on the assumption that stochastic noise in signalling and gene expression arise from a combination of many factors. Computing the single-cell channel capacity led to a result of 3.14 bits. It shows that an ‘average cell’ at 205 min after pheromone stimulation has sufficiently precise signalling to distinguish between almost nine different pheromone concentrations.

The fact that the single-cell variation only increases as the square root of the mean implies that brighter cells have higher channel capacities. For example, the channel capacity for the same average cell but at the middle time point (number 7, at 100 min after stimulation) is 2.58 bits. Back to the final time point, cells that are one standard deviation dimmer or brighter than the ‘average cell’ have channel capacities of 2.73 and 3.38 bits, respectively.

### 3.4. Internal referencing could improve signalling accuracy

The difference between the population and single-cell channel capacities is equivalent to the difference between the formal definitions of accuracy and precision. The former represent closeness to some correct answer, whereas the latter represent measurement reproducibility. Our calculations show that individual cells have responses that may be far from the population average (low accuracy) because of high cell-to-cell variability, but that each one signals consistently over time (high precision).

This raises the question of how cells can actually benefit from their precise signalling. Given that any particular cell can only mate once, it would seem that its ability to transmit signals precisely would be wasted because it would not know how to interpret its level of induced gene expression. In more detail, a scientist who measured a single fluorescent response from a randomly chosen single cell, and who already knew all relevant population-level statistics, would only be able to use this single response value to estimate the external pheromone concentration with the accuracy of the population channel capacity, which is 1.35 bits. It seems that a cell would have the same limitations. However, yeast cells have been experimentally shown to do better than this; as mentioned above, cells are able to select mating partners accurately based on the strength of their pheromone signal [3].

One possibility is that signaling accuracy could be increased if the PRS system output could be calibrated against a reliable internal standard. In particular, expression levels of different genes tend to be correlated [25,42,47], so one might imagine that a cell could compare the expression of a pheromone-responsive gene with that of a constitutively expressed gene.

We investigated how this might increase signaling accuracy by quantifying the information that a cell would learn if its YFP fluorescence value were divided by the simultaneously measured CFP value. We computed the population channel capacity as before, now with these internally referenced fluorescence values, and found that the normalization increased the population channel capacity from 1.35 to 2.01 bits. This implied that a cell with internal referencing could accurately distinguish about 4 different pheromone concentrations with a single measurement and without prior calibration. Such cellular comparison is not wholly unreasonable, given the fact that the PRS is already able to sense the fraction of receptors bound by ligand, as opposed to only the absolute number of bound receptors [8,34]. By extension, cells could use multiple internal standards, and/or standards that correlate particularly closely with pheromone-responsive gene expression, to further improve their measurements of absolute pheromone concentrations.

### 3.5. Quantification of relative sources of variation

We divided the variation in system output into categories to better understand where it arises. To do so, we used the fact that the effects of multiple uncorrelated sources of variation can be combined by adding their squared coefficients of variation, as shown above in equation (2.6). Results are summarized in table 1.

**Table 1.** Contributions of different sources of variation. The “C–L *et al*.” column presents values from [25, p. 702]. The “Contribution” column gives percentages, relative to the total variation in the same column. Values below the horizontal line either repeat or were computed from values above the line.

variation	symbols	this work	C–L *et al*.	contribution
total measured	$\eta_{pop.}^{2}$	$0.466^{2}=0.217$	—	—
experimental noise	$\eta_{exp.}^{2}$	$0.131^{2}=0.017$	—	—
total biological	$\eta^{2}(y)$	0.200	0.17	100%
single-cell noise	$\eta^{2}(\lambda )+\eta^{2}(\gamma )=\eta_{s.c.}^{2}$	$0.035^{2}=0.0013$	—	0.6%
expression capacity	$\eta^{2}(G)$	—	0.14	82%
signaling power	$\eta^{2}(L)+\eta^{2}(\lambda )$	—	0.029	17%
expression noise	$\eta^{2}(\gamma )$	—	0.002	1.2%
expression capacity	$\eta^{2}(G)$	—	—	82%
pathway capacity	$\eta^{2}(L)$	—	—	17%
expression noise	$\eta^{2}(\gamma )$	—	—	1%
pathway noise	$\eta^{2}(\lambda )$	—	—	<1%

We started with the total measured coefficient of variation, $\eta_{\text{pop.}}=0.466$, which squares to give $\eta_{\text{pop.}}^{2}=0.217$. Also, the experimental coefficient of variation was $\eta_{\text{exp.}}=0.131$, which squares to give $\eta_{\text{exp.}}^{2}=0.017$. The ratio of these two squared values shows that 8% of the total measured variation arose from measurement error, while the remaining 92% arose from biological variation. The difference of the squared values gives the biological variation as $\eta^{2}(y)=0.200$, a result that agrees well with the value of 0.017 that we found earlier from the same data set, but using different analysis methods [25] (this prior value was found by comparing CFP and YFP expression, which decreased sensitivity to experimental error).

We further subdivided the variation using equation (2.6), but with the correlation term dropped,


(3.13) $$\begin{array}{lcr} & \eta^{2}(y)=\eta^{2}(L)+\eta^{2}(\lambda )+\eta^{2}(G)+\eta^{2}(\gamma ). & \end{array}$$

The combined noise terms, $\eta^{2}(\lambda )+\eta^{2}(\gamma )$, arise from the same variation as the single-cell noise that was investigated above. However, this is complicated by the square root relationship presented in equation (3.9). We address it by extending the notion of an ‘average cell’ that was defined above by also assuming that the pheromone dose has an intermediate value; more precisely, we assume that the cell’s YFP expression level is exactly half of what it would be with a saturating pheromone dose. From equation (3.12), this average cell’s single-cell noise coefficient of variation is $0.050/\sqrt{2}=0.035$. Squaring this then gives the single-cell noise as $\eta_{\text{s.c.}}^{2}=0.0013$. This is 0.6% of the total biological variation, implying that the remaining 99.4% of the variation arose from temporally stable cell-to-cell differences.

Our data do not allow further discrimination of the terms in equation (3.14), so table 1 also lists values that Colman-Lerner *et al*. [25] computed using correlations between CFP and YFP fluorescence. Their values are not perfectly consistent with ours, such as the facts that they didn’t account for experimental noise and that their expression noise contribution, $\eta^{2}(\gamma )$, is greater than our result for the sum of gene expression noise and pathway noise, $\eta^{2}(\gamma )+\eta^{2}(\lambda )$. Nevertheless, the two sets of results are in reasonable agreement. Combining our results with theirs, shown in the lower half of table 1, leads to estimates for each of the variation terms in equation (3.14). They show that the majority of the biological variation arose from cell-to-cell differences in expression capacity, less from pathway capacity, and very little from either expression or pathway noise.

A substantial fraction of the cell-to-cell variation in system output is simply due to the fact that some cells were arrested in G1, with one copy of each fluorescent reporter gene, and others in G2, which is after DNA duplication, so they have two copies of each gene. We quantified this contribution by returning to the bimodal fit to the normalized population distribution that is shown in figure 4D. We computed the variation for the different cell cycle states as


(3.14) $$\eta_{\text{phase}}^{2}=\eta_{\text{pop.}}^{2}\left(a_{1}\mu_{1}^{2}+a_{2}\mu_{2}^{2}\right),$$

where $a_{1}$ and $a_{2}$ are the areas of the two Gaussians that we used for the fit, which add to 1, and $\mu_{1}$ and $\mu_{2}$ are the means of the two Gaussians; the mean for the total distribution is $a_{1}\mu_{1}+a_{2}\mu_{2}=0$. Substituting in the best fit values ($a_{1}=0.72$, $\mu_{1}=0.46$, $a_{2}=0.28$, and $\mu_{2}=-1.2$) leads to $\eta_{\text{phase}}^{2}=0.13$. Comparing this with the total biological variation shows that 65% of the variation arose from cells being in different cell cycle states, and 35% from other causes. This is in reasonable agreement with our prior finding that inhibiting cell cycle progression reduced total variation by about 45% [25].

Subtracting $\eta_{\text{phase}}^{2}$ from the total biological variation leads to $\eta^{2}=0.07$; this represents the sum of the cell-to-cell variation and noise terms, as in equation (3.14), but where the cell-to-cell variation only arises from cell individuality and not from cells being in different cell cycle states. Because of this reduction in total variation, the relative contribution of the single-cell noise, $\eta_{\text{s.c.}}^{2}$, increases to 2% of the total. The remaining 98% of variation between cells that have identical genes, environments, and also cell cycle states arises from temporally stable cell individuality.

## 4. Discussion

A large fraction of cell biology research focuses on *how* cells work, but much less investigates *how well* they work. This study falls in the latter category, quantifying how well yeast cells are able to transmit information from pheromone binding to cell-surface receptors, through the pheromone response signalling system, and on to the system output of protein expression. In agreement with substantial prior work [20–22,25–29], we found that cells are able to measure and report signals with high precision, but that system output varies widely between different cells, primarily due to temporally stable differences in gene expression capacity.

Using information theory, we showed that cell-to-cell differences were large enough that knowledge of a randomly chosen single cell’s output can only convey 1.35 bits of information about the stimulus level. However, we found that each individual cell can distinguish between different stimulus levels up to a precision of about 3.4 bits. We also separated the total measured variation in system output into its components, finding that, in our data, 91% of it arose from temporally stable cell-to-cell differences, 8% arose from measurement errors, and only 1% arose from stochastic noise in both the signalling pathway and gene expression.

Several of the methods described here deserve further comment. First, our use of single-cell time-series data for estimating mutual information is a novel approach that can be applied to essentially any signalling system for which these data can be collected. In particular, it enables analysis of signalling variability for cells that cannot be restimulated with different signal levels. The sole requirements on the data set are that the cells behave independently of each other and that the response information is represented by the response strength. As a counter-example, some signalling systems may encode information in the response duration [48], which could not be quantified with the approach described here.

Second, all our results were computed from smooth curves that were fit to raw data, rather than from the raw data themselves. For example, we computed the SRV diagrams shown in figure 4 from a fit to the dose–response curve, fits to graphs of fluorescence standard deviations arising at different mean fluorescence levels, and fits to histograms of cell variation. We then computed information channel capacities from these smooth SRV diagrams. This approach was essential here because only five different pheromone concentrations were investigated experimentally; if we had used these raw data, then the channel capacity results would have been artificially capped at five distinguishable levels, which corresponds to $\log_{2}\;5=2.3$ bits [49,50]. The interpolations and extrapolations that were inherent to our fits removed these artifacts. More generally, our fits represented best estimates for the underlying cell behaviors, without suffering from statistical anomolies that arise from small sample sizes.

Third, we separated cell-to-cell variation from other noise sources using a complicated normalization procedure (equations (3.4)–(3.7)) rather than by fitting equations to the single-cell data traces. Our approach, which is novel, had the benefit of introducing only one fitting parameter to each data trace ($\tilde{\mu }(s,i)$, shown in figure 5), which preserved as much of the variation as possible; in contrast, fitting each trace with a non-linear equation would have required multiple fitting parameters, each of which would have reduced the number of degrees of freedom for the data set and thus reduced the measured variation. Additionally, this normalization approach weighted variation evenly over time ($\Delta \tilde{y}(s,i,t)$, shown in figure 5 has similar statistics over all time values), whereas fitting without normalization would have increased the weighting for deviations at longer times due to the larger fluorescence values.

Fourth, we were able to separate intrinsic and extrinsic noise contributions by considering scaling behaviors between standard deviation and mean values. In particular, the cell population data exhibited a constant coefficient of variation (yellow dots in figure 4E), which supported our determination that this variation arose almost exclusively from cell-to-cell variation. Likewise, most of the single-cell data also exhibited a constant coefficient of variation (green dots in figure 4E), which also indicated extrinsic noise. Based on this scaling behavior, we concluded that this variation arose from measurement noise. Finally, some of the single-cell data exhibited a square root relationship between standard deviation and mean values (figure 6A), from which we determined that it arose from intrinsic noise.

As mentioned above, a potential concern about our results is that our SRV diagrams were based on a population-average dose–response curve rather than single-cell dose-response curves, which differ due to the response diversity effect [23]. We did so because yeast cells can only be stimulated once, making single-cell dose-curves impossible to measure in this system. Nevertheless, it turns out that this doesn’t actually matter because the mutual information equation, equation (2.2), doesn’t depend on signal values but only on signal probabilities. Thus, the mutual information is insensitive to any rescaling of the dose axis, whether with a shift that would lead to a different EC${,}_{50}$ or with an expansion or compression that would lead to a different Hill coefficient. By extension, the channel capacity is also insensitive to the use of the population-average dose–response curve versus single-cell dose–response curves, thus alleviating this potential concern.

While the channel capacity is the correct metric for quantifying the fidelity of a signalling system, the amount of information that is actually transmitted in nature is likely to be lower [15]. This is because channel capacity computations assume the signal distribution, p(s) (see equation (2.2)), that produces the most information transfer. However, most yeast cells live with the signal distribution that actually arises from pheromone secreted from potential mates, which is presumably different from the optimal one, leading to less information transfer.

Vice versa, our calculations may underestimate the true channel capacity because they are based on snapshots of signals. That is, we used the full time-dependent single-cell data to compute statistics on amounts of variation, but we then computed each channel capacity value for a single measurement at a single time point. As with any noisy response, averaging many responses over time can improve the overall precision, so cells may take advantage of this approach [28], or more sophisticated ones [51–54], to improve their measurement precision.

This work raises the question of how cells are able to accomplish precise signalling despite both extracellular and intracellular variation. To this end, we highlight several approaches, or design patterns [55], that have been identified as being important for precise signalling. One is linear signal transmission, in which the output of each step of a signalling pathway is directly proportional to the input; linear signalling is widely conserved in cell signalling systems, including in the yeast PRS [1,56] and the mammalian EGF, Wnt and TGFβ signalling pathways [57]. It enables better information transmission than non-linear signal transmission because it avoids signal saturation, along with the concomitant increased noise sensitivity and information loss [1,2,8]. Another approach is ratiometric detection, also observed in the yeast PRS [34,58] and elsewhere [59,60], in which the number of ligand-bound proteins is compared to the number of unbound proteins to determine percent occupancy. This enables a cell to accurately determine extracellular ligand concentrations, independent of its number of receptors. Our hypothetical use of an internal concentration standard, described above, is an extension of this ratiometric detection concept. Yet another approach is fold-change detection, in which a cell determines the percent change in protein binding over time, which increases sensitivity to changes in agonist concentrations and again reduces sensitivity to the absolute number of cell-surface receptors [61–65]. Yeast uses a version of this that has been called PRESS, for pre-equilibrium sensing and signalling, which particularly improves information transmission for high pheromone concentrations [66].

A separate question concerns the biological causes of the wide cell-to-cell variation that is observed in expression capacity. We quantified its width here, showing that a population of isogenic yeast cells in identical environments had a coefficient of variation of 0.466, meaning that the standard deviation across the population is almost half as large as the mean value. A substantial fraction of this variation turned out to arise from cells being in different growth phases, but removing those differences still leaves a coefficient of variation of 0.26 (from §3.5, subtracting $\eta_{\text{phase}}^{2}$ from $\eta_{y}^{2}$ yields $\eta^{2}=0.07$; taking the square root gives η=0.26). In other words, isogenic cells in identical environments and the same growth phase have response standard deviations that are 26% of the response means. Nearly all of this cell-to-cell variation appears to affect all genes equally (leading to its appellation ‘expression capacity’), suggesting that the source of the variation could be in the gene expression system. For example, stochastic variation in the numbers of ribosomes in different cells would affect all genes equally. However, this possibility does not agree with the amount of observed variation because there are about 200 000 ribosomes per cell [67] and variation for Poisson distributions, which is likely to be a good approximation in this case, is the square root of the mean, leading to a predicted variation of only about 0.2%, not the observed 26%. Alternatively, cell-to-cell variation could arise from some common upstream transcriptional regulator [43]. If so, then we can ask how many molecules of this regulator, on average, would create the observed variation? The answer is about 15 molecules ($\sqrt{15}/15\approx 26\%$), but we are unaware of any essential proteins in yeast cells with such low copy numbers. This shows that if the observed cell-to-cell variation arises from stochasticity in a single molecular species, then it is almost certainly amplified through some non-linear process, such as positive feedback. Alternatively, the variation could arise from low copy numbers for multiple different molecular species, but all of these would need to be upstream of the gene expression system. We suggest that this would be an interesting topic for further research.

Our finding that a single cell can sense, transmit signals, and respond precisely agrees with many experimental results with different types of eukaryotic cells [3,21,68–70]. These present the consistent picture that a wide variety of individual cells are able to make well-informed decisions on their own.

## Figures and Tables

**Figure 7.**
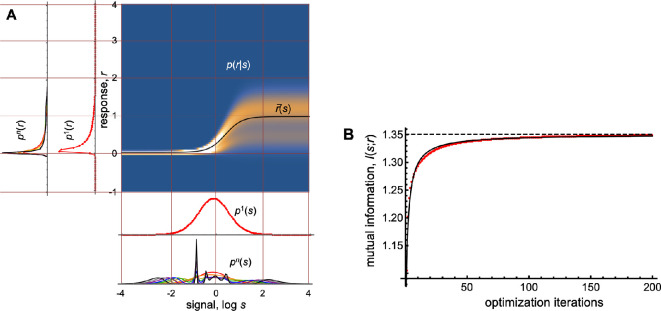
(A)The shaded region illustrates the conditional probility p(r|s) with brighter regions corresponding to higher probabilities. The x-axis represents the signal, shown with a log scale, and the y axis represents the response. Red curves below and on the left show the initial signal and response distributions, $p^{1}(s)$ and $p^{1}(r)$. Colored curves below and left of those show the evolution of the signal and response distributions over optimization iterations, $p^{n}(s)$ and $p^{n}(r)$; lines represent iterations 1 (red), 2 (orange), 5 (yellow), 10 (green), 20 (blue), 50 (magenta), 100 (purple), and 200 (black). (B) Mutual information increase over optimization iterations, showing data in red and a rational function fit from A 7 in black.

## Data Availability

All relevant data and data processing details are presented in the electronic supplementary materials, in the main text of the paper, or in the paper’s appendix [71].

## Appendix A

This appendix describes how we computed channel capacity values from the equations that underlie SRV diagrams (Mathematica source files are in the electronic supplementary material).

We started by defining the initial signal distribution as

(A 1)
$$\begin{array}{lcr} & p^{1}(s)=\frac{N}{\sigma (s)}\frac{d\bar{r}(s)}{ds}. & \end{array}$$

Here, σ(s) is the standard deviation of the variation as a function of the signal. If the coefficient of variation is assumed to be constant, then it equals $\eta \bar{r}(s)$, where η is the coefficient of variation and $\bar{r}(s)$ is the mean dose-response function; alternatively, for square root noise, it is proportional to $\sqrt{\bar{r}(s)}$ (the constant of proportionality was found in equation (3.12) to be 0.050, although this isn’t important due to normalization). The N parameter is a normalization constant set so that

(A 2)
$$\begin{array}{lcr} & \int_{s}p^{1}(s)ds=1. & \end{array}$$

To explain equation (A 1), the second factor makes the probability proportional to the slope of the dose-response curve because this leads to a uniform probability distribution over the responses. This procedure, known as histogram equalization in digital image processing and gain control in neuroscience, optimizes the information transfer in the case of uniform noise [72,73]. The first factor further weights the probability distribution in inverse proportion to the amount of noise in order to favor those signals that are subject to lower noise. The resulting initial signal distribution is optimal, meaning the one that gives the greatest possible mutual information, for cases in which the variation is much less than the mean response [9]. This condition is not actually true but nevertheless leads to a good initial estimate. Combining this signal distribution assumption with the conditional response probability, given as p(r|s) and illustrated with shading in a SRV diagram, enables calculation of the initial response distribution as

(A 3)
$$\begin{array}{lcr} & p^{1}(r)=\int_{s}p(r|s)p^{1}(s)ds. & \end{array}$$

Next, we iteratively optimized the signal distribution using the Blahut-Arimoto algorithm, which is a variational optimization of the mutual information [9]. Its procedure involves the alternation of two calculations. First, the signal distribution is updated from iteration n to n+1 with the equation

(A 4)
$$\begin{array}{lcr} & p^{n+1}(s)=\frac{1}{Z}\exp\left[\int_{r}p(r|s)\;\ln\;\frac{p(r|s)p^{n}(s)}{p^{n}(r)}dr\right], & \end{array}$$

where Z is a normalization constant, defined so that

(A 5)
$$\begin{array}{lcr} & \int_{s}p^{n+1}(s)ds=1. & \end{array}$$

Second, the response distribution is updated using a modification of equation (A 3),

(A 6)
$$\begin{array}{lcr} & p^{n+1}(r)=\int_{s}p(r|s)p^{n+1}(s)ds. & \end{array}$$

At any point, the mutual information [74] can be computed from equation (2.2). It is possible to constrain the signal distribution during the optimization, such as to add a cost to particular signals or to enforce a certain degree of smoothness [9]. However, we did not do so in this work because we sought the global optimum, without artificial constraints.

These integrals need to be computed numerically, which we did with the Mathematica software. We partitioned signal values from $10^{-4}$ to $10^{4}$ nM of pheromone with a logarithmic step size of 0.05 (i.e. possible signal values were at $10^{-4}$, $10^{-3.95}$, $10^{-3.9}$, ..., $10^{3.95}$, $10^{4}$), yielding equally spaced values along a logarithmic axis. We also partitioned response values, scaled so that $\bar{r}(s)$ approaches 1 for large s, from −1 to 4 in linear steps of 0.05. Negative response values are physically nonsensical but are mathematically necessary because the assumption of Gaussian variation makes negative responses possible. Figure 7A illustrates the discretized functions, showing p(r|s) as shading, $p^{1}(s)$ as the lower red curve, and $p^{1}(r)$ as the red curve on the left.

We optimized the signal distribution using discrete versions of the above equations:

(A 7)pn+1(s)=1Zexp⁡[∑rp(r|s)ln⁡p(r|s)pn(s)pn(r)](A 8)∑spn+1(s)=1(A 9)pn+1(r)=∑sp(r|s)pn+1(s)(A 10)I(s;r)=∑r∑sp(r|s)p(s)log2⁡p(r|s)p(r).

We performed 200 iterations of optimization, at which point the mutual information had nearly stopped increasing, shown with red points in figure 7B. However, the mutual information was still increasing slightly, so we fit the mutual information data points to a rational function of the form

(A 11)
$$\begin{array}{lcr} & I^{n}(s;r)=c+\frac{a}{1+bn}, & \end{array}$$

where n is the iteration number and a, b, and c are fit parameters; this is shown with a black line in figure 7B. Taking this equation to the limit of infinite iterations shows that $I^{\infty }(s;r)=c$, meaning that c is the channel capacity. We quoted these values in the main text.

The signal distribution evolved substantially during the optimization, changing from a simple smooth unimodal distribution to a widely spaced and spiky distribution, as shown in the bottom graph of figure 7A. It also did not stop evolving with more iterations, but continued becoming more widely spaced and more spiky. The number of spikes did not correspond to the number of distinguishable signals. For example, the population channel capacity was only 1.35 bits, which corresponds to $2^{1.35}=2.5$ distinguishable signals, but the final signal distribution has 6 peaks in it and would likely have more if we had continued the optimization.

On the other hand, the response distribution did not change appreciably after the first several iterations, as shown on the left side of figure 7A. The fact that neither the response distribution nor the mutual information changed substantially during optimization, even as the signal distribution went from a smooth unimodal distribution to a spiky one, indicates that the precise shape of the signal distribution is not particularly important. Thus, the fact that we did not constrain the smoothness of the signal distribution during optimization is unlikely to have made a substantial difference to the computed channel capacity.
